# A Green Approach to Oil Spill Mitigation: New Hybrid Materials for Wastewater Treatment

**DOI:** 10.3390/polym16152225

**Published:** 2024-08-05

**Authors:** Irina Apostol, Maria Valentina Dinu, Narcis Anghel, Iuliana Spiridon

**Affiliations:** “Petru Poni” Institute of Macromolecular Chemistry, Grigore Ghica Vodă 41 A, 700487 Iași, Romaniavdinu@icmpp.ro (M.V.D.); anghel.narcis@icmpp.ro (N.A.)

**Keywords:** natural polymers, montmorillonite, porous materials, degraded motor oil adsorption

## Abstract

This study focuses on the development of adsorptive materials to retain degraded 5w40 motor oil. The materials were prepared using xanthan (XG) and XG esterified with acrylic acid (XGAC) as the polymeric matrix. LignoBoost lignin (LB), LB esterified with oleic (LBOL), stearic acid (LBST) and montmorillonite (CL) were added into XG and XGAC matrices to obtain the adsorbents. Adsorption experiments revealed that XG/CL/LBOL had the highest adsorption capacity at 46.80 g/g, followed by XGAC/CL at 45.73 g/g, and XG/CL at 37.58 g/g. The kinetic studies, employing the pseudo-second-order (PSO) model, indicated rapid sorption rates with a good correlation to experimental data. FTIR spectra analysis have evidenced the physical nature of adsorption process, involving interactions such as hydrogen bonding, van der Waals forces, and π–π interactions. Equilibrium data fitting to the Henry, Freundlich, and Temkin isotherm models showed that the adsorption occurs within materials diverse pore structures, enhancing oil retention. Structural parameters like density, porosity, and surface area were pivotal, with XG/CL/LBOL showing the most favorable properties for high oil adsorption. Additionally, it was found that the adsorption efficiency was influenced by the material’s morphology and the presence of chemical modifications. This comprehensive evaluation highlights the potential of these novel adsorptive materials for environmental remediation applications, offering an efficient and sustainable approach to reducing degraded motor oil pollution.

## 1. Introduction

The rapid industrialization and the widespread use of automobiles have significantly increased the environmental impact of motor oil pollution, particularly in aquatic ecosystems. The inadvertent release of degraded motor oil into waters, whether through accidental spills or improper disposal practices, poses substantial threats to aquatic life, terrestrial ecosystems, and human health [1,2,3,4]. Contaminated water is characterized by a mixture of polar and non-polar organic constituents, including volatile compounds such as benzene, toluene, and xylene (ranging from 0.39 to 35 mg/L), total organic carbon up to 1500 mg/L, dissolved hydrocarbons (2 to 565 mg/L), phenols, oxygenated organics (0.009 to 600 mg/L), and a free oil layer (0.01 to 10% *v*/*v*). It also contains metals in concentrations ranging from 1000 to 279,000 mg/L [5,6,7].

The harmful effects of degraded motor oil on aquatic ecosystems are extensive [8]. Such contamination disrupts the delicate balance of aquatic life, adversely affecting fish, invertebrates, and microorganisms. Additionally, pollutants can be transported from aquatic to terrestrial environments, leading to broader ecological ramifications. Contaminants from motor oil also have the potential to bioaccumulate within the food chain, posing additional risks to wildlife and humans [1,9].

Given the critical threats posed by motor oil pollution, there is an urgent need for effective and rapid remediation strategies. Traditional cleanup methods are often time-consuming, costly, and may not address to the complex mixture of pollutants present in used motor oil. Adsorption has emerged as a promising alternative, offering a rapid, cost-effective, and efficient means of removing contaminants from water [10,11]. Natural polymer-based sorbent materials have gained significant interest due to their biodegradability, inherent reliability, availability, and cost-effectiveness. Functional groups such as C–O, O–H, and C=O in these polymers play a crucial role in oil adsorption [9,12].

Combining organic and inorganic compounds can enhance the adsorptive capabilities of materials. Inorganic materials, particularly clays like montmorillonite, provide advantages such as rapid adsorption kinetics and high capacity. Montmorillonite, a layered mineral clay, has surface features that make it an effective sorbent for wastewater treatment [13]. However, while many natural sorbents can retain substantial amounts of oil, their physical, chemical, and mechanical stability is often limited. Research has shown that integrating natural polymers with montmorillonite creates new systems that are more robust and effective in managing used motor oil pollution [14,15].

This paper addresses the need to reduce the environmental impact of used motor oil pollution by investigating the potential of adsorptive materials based on natural polymers and montmorillonite. By exploring the adsorption capabilities of these materials, this study aims to provide a swift and practical solution to the pressing environmental challenge posed by motor oil pollution in water.

## 2. Materials and Methods

### 2.1. Reagents

LB was obtained through the LignoBoost process from softwood. Oleic (OL) and stearic (ST) acids, along with dimethylformamide (DMF), were procured from Sigma-Aldrich (USA). XG (molecular weight = 2.5 × 10^6^ Da), obtained from *X. campestris*, and lipase sourced from CP Kelco US (Atlanta, GA, USA) and Sigma-Aldrich (Burlington, MA, USA), respectively were used without further modification. Ethanol and ethylic ether were obtained from Merck & Co. (Rahway, NJ, USA). Shelsite 30B montmorillonite nanoparticles (CL), characterized by a purity of 99%, APS < 80 nm, density of 2.8 g/cm^3^, and molecular weight of 202.185 g/mol, were procured from Nanoshel LLC (Wilmington, DE, USA) and used in their as-received state. Quaternary ammonium groups cover the CL surface (according to the Specification Sheet) making it hydrophobic. 5w40 motor oil was purchased from a local market. It underwent intentional prior usage and degradation under real-use conditions for one year.

### 2.2. Lignin Esterification

Esterification of LB with OL and ST acids was carried out using a method described in a previous study [16]. The nomenclature for the resulting lignin esters was assigned according to the fatty acid used, resulting in designations such as LBOL (LB with oleic acid), and LBST (LB with stearic acid).

### 2.3. Xanthan Esterification

The esterification of XG with acrylic acid was carried out using a previously described method [17] in order to confer hydrophobic properties.

### 2.4. Adsorptive Materials Obtainment

Adsorptive materials were prepared using a method outlined in a prior research study [13]. For that, CL, LB or its esters were added into XG or XGAC, which were used as the polymeric matrix (Table 1). The resulting materials were obtained through a series of 10 freeze–thaw cycles followed by lyophilization.

### 2.5. Adsorption of Degraded Motor Oil

In a 50 mL beaker, various amounts of degraded motor oil (1 g, 2 g, or 3 g) were mixed with 25 mL of water. The materials were weighted before immersing them into the oil–water systems. After a 120 s time interval, the samples were extracted from the systems. The excess of oil present on the material surface was removed using filter paper, and subsequently, the samples were re-weighted. The sorption experiments were performed at 25 °C. The sorption capacity (q_e_, g/g), the quantity of modified oil retained within a specific time interval (q_t_, g/g), and the efficacy of used oil removal (R%), were detailed in the Supplementary Material, Equations (S1)–(S3).

The adsorption volume factor (f) was calculated according to the below equation, to enhance our comprehension of the degraded motor oil adsorption process within the material pores.

$$f=\frac{v_{oil}}{v_{pores}}=\frac{\frac{m_{oil}}{\rho_{oil}}}{\frac{m_{s}}{\rho_{pb}}\times \varepsilon_{p}}$$

where *v*_oil_ and *v*_pores_ are the degraded oil sorption volume (cm^3^) and pore volume of material (cm^3^), respectively. *m*_oil_ and *ρ*_oil_ are the mass (g) and the density of adsorbed altered oil (g/cm^3^). *m*_s_, ρ_pb_, and ε_p_ are the mass (g), the density (g/cm^3^), and porosity (%) of material.

### 2.6. Adsorption Kinetics

Investigating the kinetics of adsorption provides critical insights into the rate at which degraded oil is captured by the adsorbent. Understanding this kinetics is essential for optimizing operational conditions in industrial batch processes. To achieve this, samples were weighted at specified time intervals (1, 3, 7, 60, and 120 s). To deepen the understanding of the adsorption kinetics and mechanisms, the pseudo-first-order (PFO) and pseudo-second-order (PSO) kinetic models were used. The corresponding linear Equations (S4) and (S5) are detailed in the Supplementary Material. The alignment between the mathematical models and the empirical data was assessed by the examination of the χ^2^, Δq, and RMSE values (Equations (S6)–(S8)).

### 2.7. Isotherms Analysis

The dynamic interaction between the materials and modified oils, in varying quantities of 1 g, 1.5 g, 2 g, 2.5 g, and 3 g in 25 mL distilled water was analyzed using adsorption isotherms. These isotherms provided insights about the equilibrium distribution of oil molecules between the liquid and solid phases. To deepen the understanding of the materials’ adsorption characteristics, several isotherm models were used, including Henry, Langmuir, Freundlich, and Temkin (see Supplementary Material). By fitting the experimental adsorption data to these models, it was identified the most appropriate model for each experiment, based on the R^2^ value.

### 2.8. Regeneration and Reusability Evaluation

For the regeneration of produced adsorbents, and to investigate their reusability after the sorption process, the oil ladened sorbents were compressed between two flat plates, to desorb the oil. They were then rinsed in n-hexane and in deionized water, and subsequently placed in an oven maintained at 60 °C until dryness. After drying, the regenerated materials were reused until a structural collapse was observed.

### 2.9. The Characterization of Materials

The density and porosity of the materials were determined according to a method described in a previous study [13].

Surface morphology analysis was performed using a VEGA TESCAN microscope. The samples were fixed onto aluminum stubs using carbon adhesive disks, and their surfaces were examined utilizing a low-vacuum secondary electron detector at an acceleration voltage of 20 kV. All analyses were performed at room temperature.

The dynamic water vapor sorption capacity of the materials under investigation was determined using a fully automated gravimetric analyzer, IGAsorp, provided by Hiden Analytical (Warrington, UK). Weights changes were recorded as a function of relative humidity (RH) within a range of 0–90%, with 10% increments, at a constant temperature of 25 °C. Additionally, based on the isothermal studies, the specific surface area was computed as it was presented in a previous work [13].

Zeta potential was determined on a Malvern Panalytical Zeta-sizer Advance Pro Red (Malvern Panalytical Ltd., Malvern, UK), at a constant temperature of 25 °C.

Compression tests were undertaken using a Shimadzu Testing Machine (EZ-LX/EZ-SX Series, Kyoto, Japan). The compressive stress (σ, kPa), strain (ε), and elastic modulus (G, kPa) were assessed for all the ethanol-swollen samples (18 mm width, 3 mm height, and 10 mm thickness) following established protocols previously outlined for other macroporous materials [18,19,20].

FTIR spectra of the materials were recorded, both before and after the adsorption experiments using a Vertex 70 FTIR spectrometer from Brüker. The spectrometer was equipped with an ATR (Attenuated Total Reflectance) device with a ZnSe crystal. Maintaining a spectral resolution of 2 cm^−1^, measurements were carried out at a 45-degree angle of incidence, covering the range of 4000–600 cm^−1^.

## 3. Results and Discussion

### 3.1. The Adsorption Capacity of Degraded Motor Oil

One of the pivotal features of an adsorptive material lies in its adsorption capacity, representing a key determinant of its effectiveness in capturing and retaining target substances. As it was already mentioned, this study focuses on degraded 5w40 motor oil retention. This kind of degraded oil represents a dangerous pollutant, having a TAN (Total Acid Number) value of 2.13 mg KOH/g (unused motor oil typically has a TAN value lower than 2 mg KOH/g) [21].

The adsorption capacities of all the obtained materials for different initial amounts of oil are presented in Table 2. The evaluation of adsorption capacities unveils a ranking among the tested materials, where XG/CL/LBOL demonstrates the highest effectiveness, succeeded by XGAC/CL, XG/CL, XGAC/CL/LBST, XG/CL/LBST, XGAC/CL/LBOL, XG/CL/LB, and XGAC/CL/LB. This emphasizes the differences in their individual adsorptive performances.

**Figure 1:**
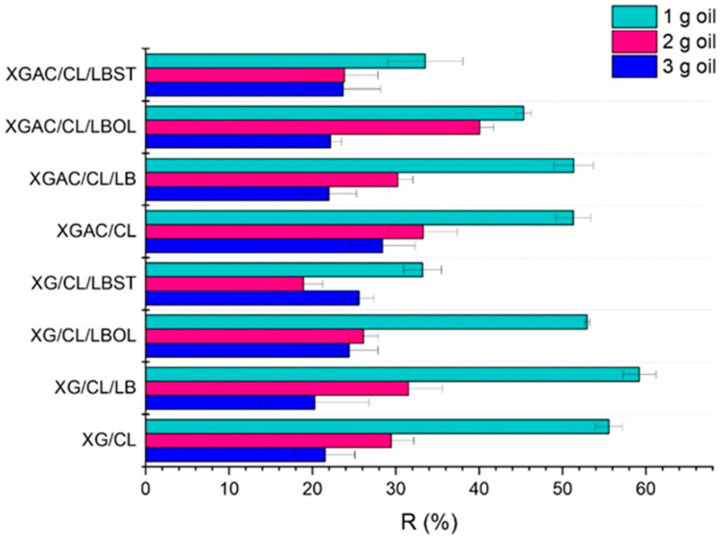
The efficacy of used oil removal.

The amount of motor oil within the oil–water system directly impacts the adsorption capacity of the materials, exhibiting an increase in adsorption capacity with the rise dosage of motor oil from 1 to 3 g. Equilibrium adsorption was achieved within 120 s. The R (%) values of the materials also vary depending on the quantity of oil present in the systems. According to Figure 1, R (%) values decrease with increasing oil amount.

The percentage of oil removal decreases, while the adsorption capacity increases, as the initial oil concentration rises. This could be because, at higher concentrations, the adsorption surface and active sites are completely filled (adsorbent saturation), which is not achievable at lower concentrations [22].

Our materials presented different sorption capacity, as function of materials formulation, the highest sorption capacity (46.8 g/g) being recorded for XG/CL/LBOL system. Almost all materials show sorption capacity values which are still higher than that of conventional PP fibers or that of zeolites derived from fly ash (0.36–0.91 g/g) [23], zeolitic imidazolate and polycaprolactone nanocomposites (12.1 g/g) [24], silica aerogels (9.56 g/g) [25] or non-woven materials based on recycled jute fibers (9.81–16.53 g/g) [26].

### 3.2. The Impact of Chemical Modification of XG and LB upon the Adsorption Capacity

A commonly employed technique for enhancing oil adsorptive properties involves chemical modification [27,28]. This method involves converting the functional groups attached to organic materials into hydrophobic groups, leading to the enhanced sorption capacity of materials. The esterification of XG and LB introduces new chemical groups to their structures, enhancing their affinity for oil molecules by hydrophobic interactions [29]. Through esterification, XGAC/CL material retain a higher amount of motor oil (45.73 g/g) as compared with material based on unmodified XG, XG/CL, which retain 39.58 g/g of degraded motor oil. This was also true for chemically modified lignin. For example, XG/CL/LBOL retained the highest amount of degrade oil (46.80 g/g), as compared with other materials due to the presence of LB esterified with OL.

### 3.3. The Effect of CL on Adsorption Capacity of Materials

The use of CL modified with quaternary ammonium groups has been investigated to elucidate its effect on the adsorption properties of the materials. The introduction of quaternary ammonium groups slightly decreased the adsorption capacity (as compared with data from another study [16]), yet concurrently, it significantly reinforced the structural integrity of the materials [30,31]. This fortification renders the materials more robust and easier to handle in practical applications by avoiding the network failure from water. It showcases a balance between adsorption efficiency and enhanced mechanical properties (as will be shown in Section 3.4.4). The reinforced materials exhibit improved resistance to physical stress, making them more convenient for various handling scenarios, thereby broadening their potential utility in real-world environmental remediation efforts.

### 3.4. The Impact of the Structural Properties of Materials on Their Adsorption Capacity

The degraded motor oil adsorption process is also influenced by the structural characteristics of the adsorptive material. The inherent features of the adsorptive materials play a pivotal role on its efficacy in adsorbing oil. Porous structures facilitate increased surface interaction [32], while optimal density ensures efficient oil retention. The zeta potential value of adsorptive material additives is vital as it influences particle stability, directly affecting the efficiency of motor oil adsorption. Surface area governs the available space for oil molecules to be adsorbed, and compression resistance ensures the material’s stability under external forces.

#### 3.4.1. The Effect of Materials’ Density and Porosity on the Adsorption Capacity

The link between density and porosity is complex, and finding an optimal balance is crucial for achieving materials with enhanced motor oil adsorption properties. This interdependence highlights the significance of refining material characteristics for optimal performance in oil retention applications.

Table 3 shows the values of density and porosity of materials based on XGAC. The materials exhibit a combination of low density and high porosity, rendering them well-suited for conducting oil adsorption experiments. Materials comprising XGAC and LB esters present a higher density (0.064 and 0.068 g/cm^3^) due to the formation of numerous intramolecular interactions between long chain alkyl groups of LBOL or LBST and the polymeric matrix. In contrast to OA, SA possesses a linear structure capable of establishing numerous intermolecular hydrophobic interactions. Consequently, the system incorporating LBST exhibits higher density (0.068 g/cm^3^) and lower porosity (72.94%). Conversely, the molecular structure of OA results in fewer intermolecular interactions, aligning with expectations and causing a slight decrease in density (0.064 g/cm^3^), and an increase in the porosity (96.56%) of the XGAC/CL/LBOL material.

Regarding adsorption capacity, it could be observed that XG/CL/LBOL material (which present the lowest density—0.021 g/cm^3^ and the highest porosity—98.25%) retain the highest amount of degraded motor oil.

**Figure 2:**
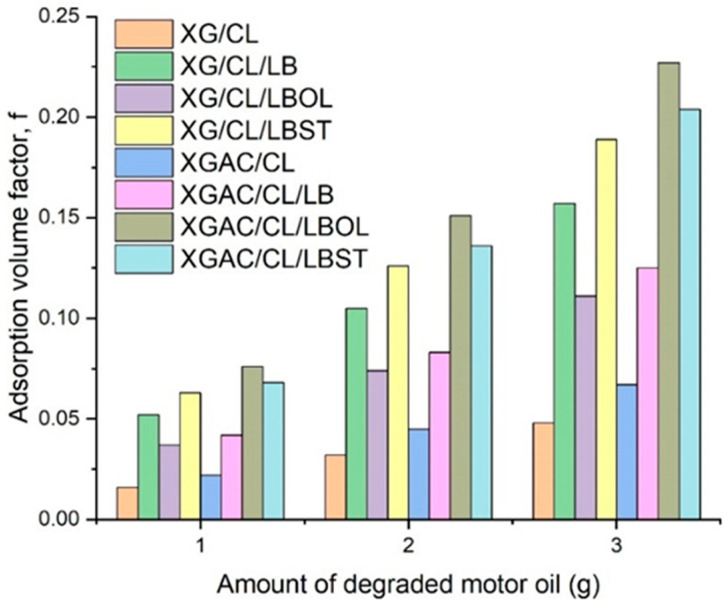
Adsorption volume factor values of different degraded motor oil amounts.

Figure 2 displays the f factor values for each material, revealing a consistent range across different oil amounts. This underlines the significance of pore volume in interpreting oil adsorption capacities. The focus should be on maximizing the accessible pore volume. Interestingly, higher adsorption capacities do not consistently correlate with greater porosity. As the oil quantity decreases, there are fewer oil molecules present to interact with the material’s active sites, resulting in reduced adsorption volume factor. This indicates the presence of less accessible pores in the materials and also suggests that some pores may be blocked or too narrow for oil molecules to penetrate effectively. Consequently, the materials exhibit decreased oil adsorption efficiency due to the restricted accessibility of these pores, emphasizing the importance of maximizing pore accessibility for enhanced adsorption performance.

#### 3.4.2. The Impact of Materials’ Morphology upon Their Adsorption Capacity

**Figure 3:**
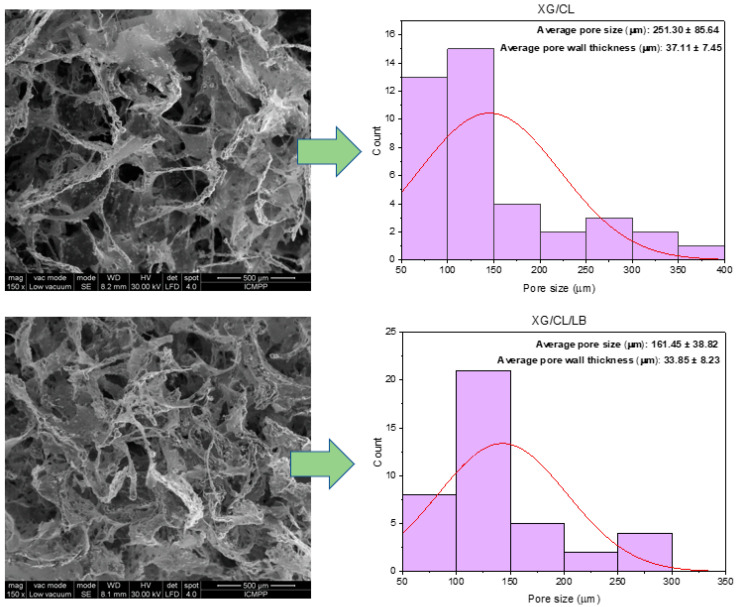

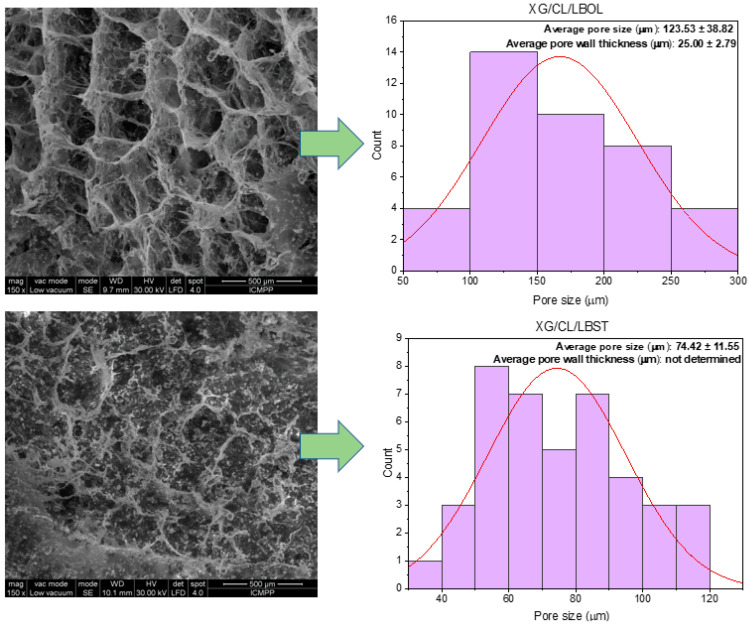
SEM micrographs and pore size distribution of materials based on XG.

**Figure 4:**
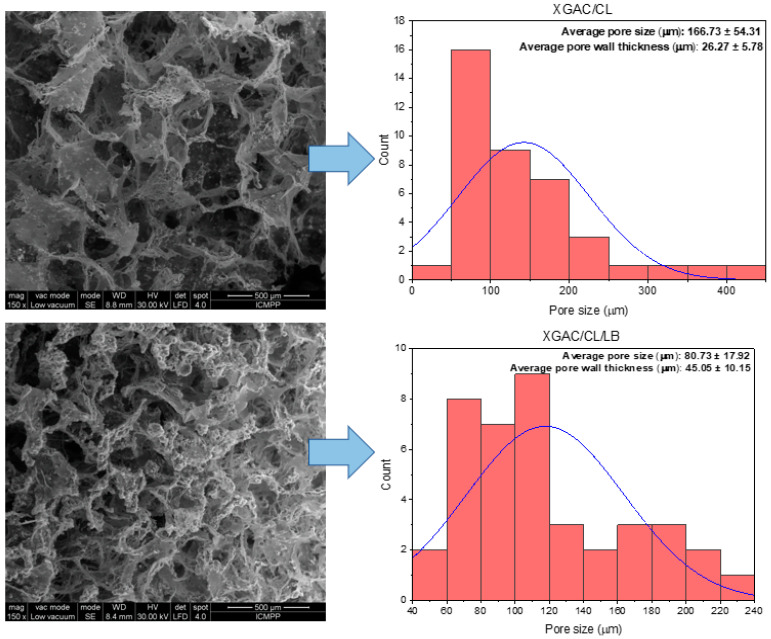

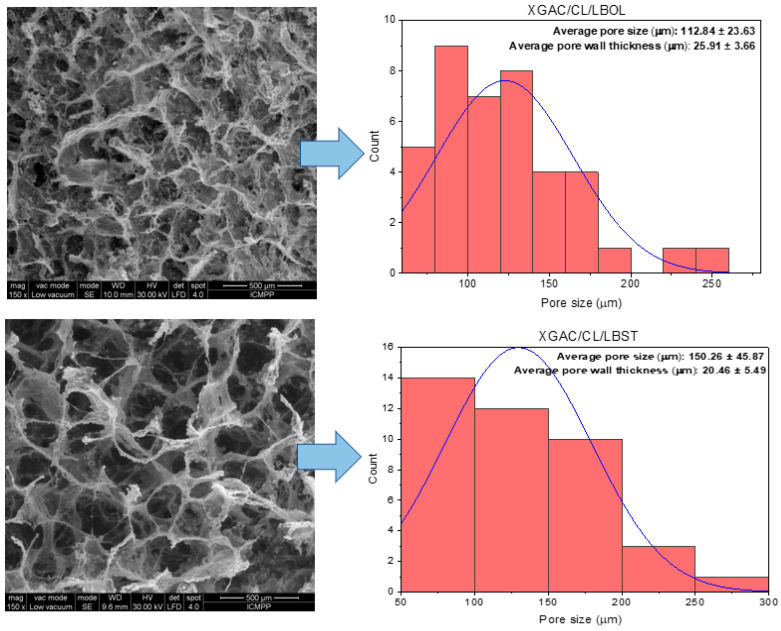
SEM micrographs and pore size distribution of materials based on XGAC.

Scanning electron microscopy (SEM) enables the characterization of sample attributes, including surface roughness, internal pores, hollow structures, and porosity. The chemical composition, physical configuration, and molecular arrangement of the material are factors influencing its ability to retain oil. Materials characterized by pores and holes of diverse sizes, displays porous interior, a morphology that intensifies with increased porosity. Throughout the adsorption process, capillary action facilitates the penetration of oil into the porous and hollow structure of the material. Consequently, the primary mechanism for degraded motor oil sorption in this sorbent involves adsorption, wherein oil molecules are entrapped by the rough surface and subsequently transferred into the voids and pore structure of the sorbent via capillary action. SEM micrographs of the studied materials are presented in Figure 3 and Figure 4. Pore sizes and pore wall thicknesses were measured from SEM micrographs using ImageJ software.

The degraded motor oil retention capacity of the studied materials was significantly influenced by their pore size, pore wall thickness, and pore size distribution. Larger pore sizes, exemplified by the XG/CL/LBOL (123.53 µm), XG/CL (251.30 µm) or XGAC/CL (166.73 µm) materials, facilitate greater fluid uptake due to increased space within the pores, resulting in the highest retention of degraded motor oil. In contrast, the XGAC/CL/LB material, for example, with a smaller average pore size of 80.30 µm, retains the lowest amount of oil, as smaller pores restrict fluid volume. Material wall thickness further impacts retention, with thinner walls, such as the 25.00 µm thickness in XG/CL/LBOL, enhancing fluid flow and interconnectedness of the pore network, thereby improving oil retention. Conversely, the thicker walls in XGAC/CL/LB (45.05 µm) hinder fluid flow and reduce retention capacity. Additionally, a broad pore size distribution, which includes a mix of small and large pores, can optimize the retention of various fluid types by maximizing the available pore space and facilitating efficient infiltration and distribution [33]. This is evident in XG/CL/LBOL, where the combination of larger average pore size, thinner walls, and favorable pore size distribution synergistically enhances its retention capacity compared to XGAC/CL/LB material.

#### 3.4.3. The Effect of Materials’ Surface Area on Their Adsorption Capacity

One key factor influencing the motor oil adsorption capacity is the surface area of the material. Generally, a material with a higher surface area can adsorb more motor oil because it provides more space for the oil to adhere to. Understanding the role of surface area in oil adsorption can lead to the development of more effective materials for oil management and environmental protection. DVS data of materials based on XGAC are presented in Table 4. Those of materials comprising XG are presented in a published paper [13].

The comparison of specific surface areas among the studied materials revealed no statistically significant differences, attributed to the presence of CL, which may form well-organized structures within the polymeric matrices. This suggests that the structural organization imparted by CL does not substantially influence the overall specific surface area. Minor variations observed between the specific surface areas of the different polymeric matrices, namely XG and XGAC, could be attributed to the incorporation of LB or its esters. This minor deviation indicates that presence of LB or its esters may induce slight changes in the material’s porosity (above discussed) and, consequently, its specific surface area. Interestingly, a positive correlation was observed between the specific surface area of the materials and their capacity to retain motor oil. XGAC/CL, XG/CL/LBOL and XG/CL, which retain the highest amounts of motor oil, present the highest surface area (172.80 ± 6.27 m^2^/g, 170.44 ± 2.54 m^2^/g and 169.25 ± 1.17 m^2^/g) and sorption capacity (24.37 ± 1.45% d.b., 23.91 ± 3.77% d.b. and 25.59 ± 2.00% d.b.). This suggests that the increased surface area provides more available sites for oil adsorption, enhancing the material’s overall oil retention capacity.

#### 3.4.4. The Impact of the Materials’ Compression Resistance upon Their Adsorption Capacity

The compressive mechanical characteristics of the materials based on XGAC/CL were examined to evaluate how the composition of the materials’ network affects the elasticity and toughness (mechanical characteristics of the materials based on XG/CL were discussed in a previous study [13]). The recorded values for compressive nominal stress, strain, and compressive elastic modulus can be found in Table 5.

In the case of the materials containing as main matrix XGAC, the σ-ε profiles comprised also of two regions (Figure S1A) as for the unmodified XG networks (discussed in another published paper [13]). Thus, a first linear domain up to 64.93%, 78.43%, and 88.46% strain for XGAC/CL/LB, XGAC/CL/LBOL, and, respectively, XGAC/CL, and then a densification region due the gradual compression of pores was noticed, which are characteristic for highly porous materials. The XGAC/CL, XGAC/CL/LB, and XGAC/CL/LBOL materials exhibited tough and more rigid networks in comparison to XG/CL, XG/CL/LB, and XG/CL/LBOL due to the lower values of the maximum compression strains demonstrated by the former ones. These results are also sustained by the higher values of the elastic moduli for XGAC/CL (10.29 kPa), XGAC/CL/LB (7.34 kPa), and XGAC/CL/LBOL (5.11 kPa) materials (Figure S1D) as compared to those of XG/CL (4.35 kPa), XGAC/CL/LB (1.69 kPa), and XGAC/CL/LBOL (3.19 kPa) materials. This behavior is further supported by the changes occurred in the internal morphology (average pore sizes, and wall thicknesses of the composites before and after XG modification—Figure 3 and Figure 4).

#### 3.4.5. The Effect of Additives Zeta Potential upon Materials Adsorptive Properties

Zeta potential is a key indicator of the surface charge of particles [34] and their interaction with contaminants and stability in suspension [35]. The zeta potential values of the additives used in this study varied (see Table 6), providing insights into their impact on the adsorption process. The larger the absolute value of the potential, the more stable the adsorption [36].

The zeta potential of CL results in effective adsorption due to electrostatic attraction, but its moderate value may limit the extent of dispersion within the matrix, potentially leading to some aggregation. Also, the increased rigidity of the materials is likely due to the electrostatic charge of CL, which forms electrostatics or van der Waals bonds [33] with the negatively charged LB or LB esters.

LB exhibited the highest zeta potential (−31.06 ± 0.7 mV) among the fillers, indicating strong negatively surface charge. This high zeta potential enhances electrostatic attraction with the motor oil, improving adsorption efficiency. Additionally, the high zeta potential aids in maintaining a stable dispersion within the matrix, preventing aggregation and ensuring maximum surface area exposure for adsorption [37].

Functionalization or chemical modifications of compounds surfaces have induced some changes in zeta potential value [37]. Thus, the esterification of LB with OL and ST acids decreased the zeta potential of the resulting esters. With a zeta potential of −21.67 ± 0.9 mV, LBOL provides a good balance between electrostatic attraction and dispersion stability. From this reason, the materials which present the highest adsorption capacity also contain LBOL as additive. The moderately high negative charge facilitates effective adsorption of motor oil while maintaining good dispersion within the matrix, optimizing the adsorption capacity.

The relatively lower zeta potential of LBST (−14.19 ± 0.06 mV) indicates weaker electrostatic interactions with motor oil compared to the other fillers. While still providing some degree of attraction, the lower zeta potential may result in less efficient adsorption and potential aggregation within the matrix [37], reducing the overall surface area available for adsorption. Thus, materials comprising LBST present moderate degraded motor oil adsorption capacity (36.70 ± 1.78 g/g, 39.31 ± 4.42 g/g, respectively).

### 3.5. The Influence of Experimental Parameters upon Materials Adsorptive Capacity

Contact time and the quantity of degraded oil, significantly impact the adsorption process. The duration of contact establishes the equilibrium state, while the amount of oil determines the saturation level of the material. The influence of these parameters will be presented upon in the following sections.

#### 3.5.1. The Effect of Contact Time upon Adsorption Process (Kinetic Study)

Figure S2a,b present plots depicting the PFO and PSO kinetics for the sorption of degraded 5w40 motor oil onto materials. Upon analyzing the linearized relationship of the two kinetic models, the experimental data exhibit a superior fit (R^2^ > 0.9560) to the PSO model. The corresponding results are detailed in Table 7. Additionally, the low values determined for Δq, χ^2^, and RMSE (refer to Tables S1 and S2 in the Supplementary Materials) indicate that degraded motor oil adsorption aligns well with the PSO model.

The main mechanism driving sorption involves the initial coating of the sorbent surface via adhesion and cohesion, resulting in quick oil adsorption. Following this, secondary factors likely involve adsorption, where effectiveness is significantly influenced by chemical treatments (previously discussed) [29]. The sorption of oil onto the sorbents aligns well with the PSO kinetics, a finding consistent with other reports [13,38,39,40]. The PSO kinetic model is grounded in the concept that the rate-controlling step in the adsorption system is chemical adsorption, offering predictive insights into the system’s behavior across the entire range of contact times.

**Figure 5:**
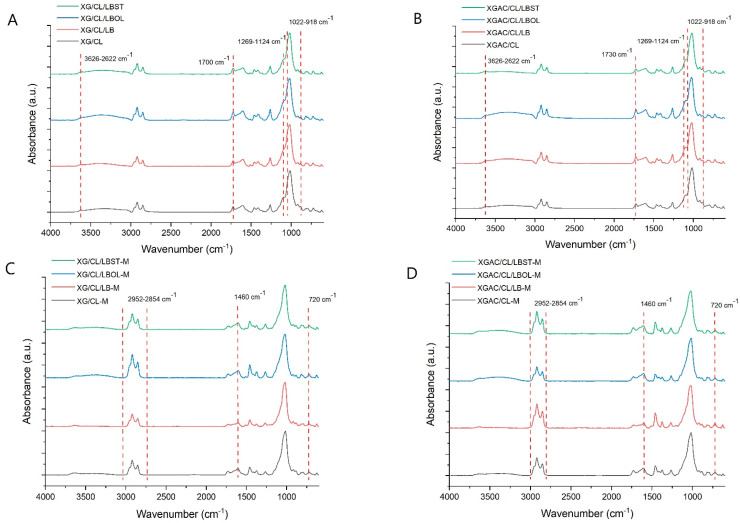
FTIR spectra of materials based on (**A**) XG and (**B**) XGAC before adsorption experiments of degraded motor oil adsorption experiments; FTIR spectra of materials based on (**C**) XG and (**D**) XGAC after adsorption experiments of degraded motor oil.

The FTIR spectra (Figure 5) of the materials before and after adsorption of degraded motor oil were systematically recorded to discern the nature of the process. The signals from the region between 3622 and 3626 cm^−1^ are ascribed to the structural –OH group within the CL structure [41,42]. Signals around 1700 cm^−1^ are assigned to the C=O and –COO^−^ groups present in XG structure while the signal from 1730 cm^−1^ is due to the presence of ester group from XGAC [43,44]. The absorption bands from the region 1022–920 cm^−1^ evidence the presence of Si–O–Si bonds and Al_2_OH bending from CL structure [42]. After adsorption process, FTIR spectra (Figure 5C,D) present new signals attributed to the oils structure. The development of hydrophobic interactions between the materials and motor oil is evidenced by the heightened intensity of signals corresponding to C–H bonds (2952–2854 cm^−1^) in the FTIR spectra of the oil-loaded materials. In the course of the internal combustion engine’s operation, engine oils are subjected to elevated temperatures and pressures, oxygen exposure, and contact with diverse metals. Consequently, hydrocarbons within the oil undergo processes of oxidation, condensation, and decomposition [45]. Absorption bands found at 720 cm^−1^ corresponds to the valence vibrations of the peroxide groups (–C–O–O–) exhibiting heightened chemical activity [46]. These groups are generated through the oxidation of hydrocarbons, a consequence of the operation of car engines and their signals’ intensity are decreased, resulting from the interaction between the –OH groups of adsorptive materials and the peroxide groups of degraded motor oil [47,48].

Notably, the examination of these spectra did not reveal the emergence of new chemical bonds, indicative of a lack of chemical interaction during the adsorption process. The FTIR spectra confirm that adsorption mechanism involves physical interactions, such as van der Waals forces, hydrogen bonding, or π–π interactions, rather than the formation of covalent bonds.

#### 3.5.2. The Influence of Initial Degraded Motor Oil Amount upon Adsorption Process (Adsorption Isotherms Study)

The development of adsorbents for pollutant removal in wastewater needs a profound understanding of adsorption isotherms. Those serve as valuable tools for discerning the physicochemical factors governing adsorption performance [49]. The plots of the isotherm model depicting the adsorption of degraded motor oil onto materials are illustrated in Figure S3, with the corresponding model parameters provided in Table 8.

Based on the highest linear regression (R^2^ = 0.9207–0.9656), it was found that experimental results obtained for XG/CL/LB, XG/CL/LBOL and XGAC/CL/LBOL materials correlate with the Henry isotherm model. In these cases, the adsorption process is assumed to be both linear and reversible. The fundamental mechanisms dictating these partition processes involve electrostatic interactions, van der Waals interactions, and hydrophobic interactions, as previously mentioned [50].

For the materials XG/CL/LBST, XGAC/CL and XGAC/CL/LB, experimental data are fitted to the Freundlich model (R^2^ = 0.9673–0.9947), that offers a more precise depiction of the adsorption process, taking into consideration the varied interactions taking place at different pores on the adsorbent surface. This model is suitable for investigating adsorption on surfaces that are rough and multisite (heterogeneous) [51].

The experimental results of XG/CL and XGAC/CL/LBST material are fitted to the Temkin isotherm model (R^2^ = 0.9337 and 0.9924). According to this, the adsorption process is considered a multi-layer process [51].

The surface of the adsorbent was notably non-uniform (see SEM images, Figure 3 and Figure 4), and the materials’ pores did not exclusively retain a single layer of molecules. This explanation clarifies why the Langmuir model did not align effectively with the experimental data in this study. A substantial amount of oil could be trapped within the pores. Consequently, the Henry, Freundlich and Temkin models indicated that the oil adsorption process on the materials surface was characterized by non-uniform or multi-layer sorption. A similar observation has been made by Zhu et al., for magnetic polydivinyl benzene nanofibers with heterogenous surface [52].

**Figure 6:**
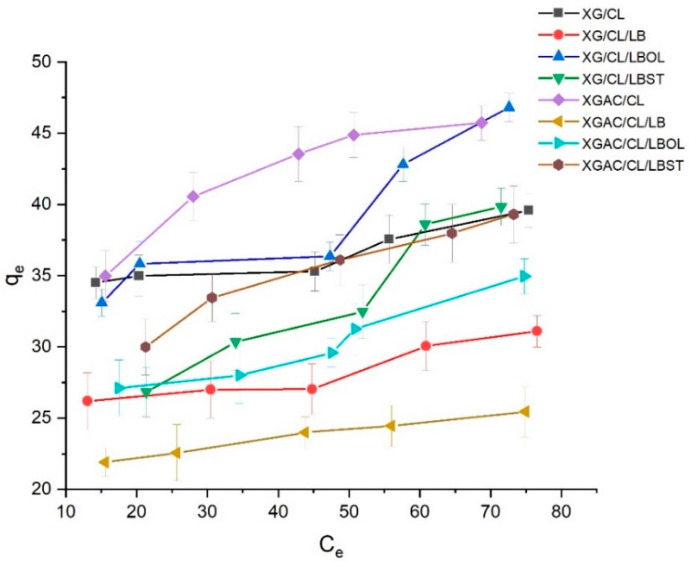
Adsorption isotherms of used motor oil by the studied materials.

The experimental adsorption profiles (Figure 6) of degraded 5w40 motor oil onto materials can be classified as “H-type”, indicating substantial solute affinity [53]. The initial part of the isotherm is vertical or, in some cases, the curve is a horizontal line running into the vertical axis suggesting that the adsorbed species are often large units (ionic micelles, fatty acids or polymeric molecules). This observation is correlated with high degraded motor oil adsorption capacities of all the studied systems.

**Figure 7:**
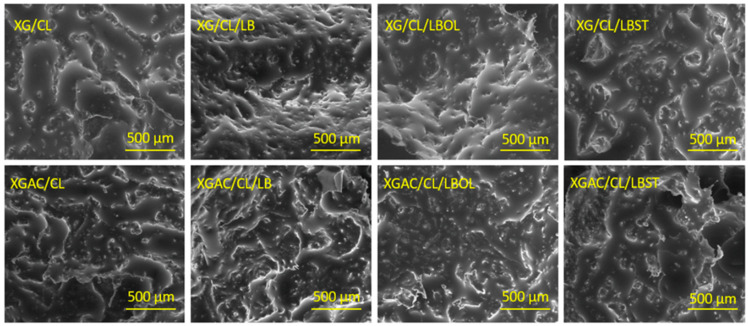
SEM micrographs of degraded motor oil loaded materials.

As further evidence of the adsorption phenomenon, Figure 7 displays SEM images of oil-loaded materials. Initially, the pores of all materials were completely empty, and upon reaching adsorption equilibrium, the pore volume became fully occupied. This behavior mirrors findings in a previous study [13]. This observation aligns with isotherm studies indicating that the adsorption process occurs within materials characterized by diverse pore shapes and sizes, influenced by various intra- or intermolecular interactions.

**Figure 8:**
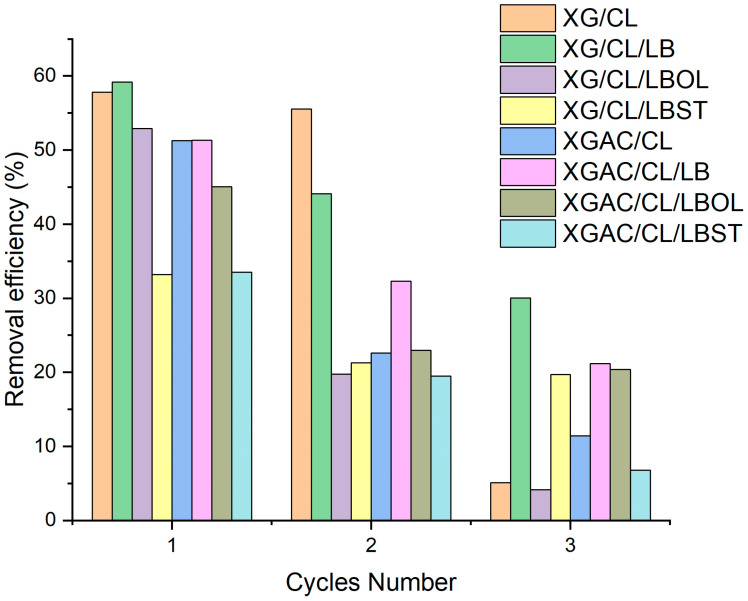
Repeatability and regeneration adsorption performance of materials.

The reusability of adsorbents is a serious factor to consider in a bid for waste minimization. The reusability of the materials was assessed through three cycle tests (Figure 8).

After the first sorption cycle, XG/CL, XG/CL/LB, XG/CL/LBOL, XGAC/CL and XGAC/CL/LB showed more that 50% oil recovery rates (57.82%, 59.18%, 52.91%, 51.28% and 51.28%), XGAC/CL/LBOL presenting an oil recovery rate of 45.03%. After the second oil sorption cycle, all materials registered a drop in oil removal rates. The sudden decrease in oil sorption capacity from the second to the third cycle could be attributed to the irreversible deterioration of the matrix due to strong mechanical pressure and residual oils trapped in the void of fibrous sorbent [54].

## 4. Conclusions

This study investigated the adsorption capacities of various materials based on XG/XGAC, LB/LB esters and CL for the remediation of wastewaters polluted with degraded 5w40 motor oil.

The highest adsorption capacity recorded was 46.80 g/g for XG/CL/LBOL, indicating superior performance in capturing and retaining degraded motor oil. The esterification of XG and LB significantly improved the materials’ oil adsorption properties. For instance, the XGAC/CL material, which includes chemically modified XG, retained 45.73 g/g of motor oil, compared to 39.58 g/g for the unmodified XG/CL. The introduction of ester groups into the polymers’ structures increased their compatibility with hydrophobic oil components, thereby enhancing their overall adsorption capacity.

Incorporating CL into the polymeric matrices also enhanced the adsorption capabilities of the composite materials. Despite a slight reduction in adsorption capacity due to the introduction of quaternary ammonium groups, the structural integrity and mechanical strength of the materials were significantly improved. This reinforcement ensures that the materials can withstand physical stress and maintain their structural integrity in aqueous environments, making them more practical for real-world applications.

The kinetic studies revealed that the adsorption process followed pseudo-second-order (PSO) kinetics. This model was supported by high correlation coefficients (R^2^ > 0.9560) and low values of Δq, χ^2^, and RMSE, which align with the PSO model’s predictions. Additionally, the adsorption isotherms fitted well with the Henry, Freundlich, and Temkin models, depending on the material, highlighting the complexity and heterogeneity of the adsorption processes involved.

The high adsorption capacities, coupled with the improved mechanical properties of the composite materials, suggest their potential for environmental applications, particularly in the remediation of oil-polluted water. The materials’ ability to rapidly reach adsorption equilibrium within 120 s demonstrates their efficiency. The regeneration of materials indicated that these could be reused for two cycles without significant loss in absorption performance. These findings contribute to the increase in knowledge on sustainable pollution management and highlight the potential of bio-based materials in overcoming urgent environmental issues.

## Figures and Tables

**Table 1:** The composition of developed materials.

Material	Composition	Mass Ratio
XG/CL	XG and CL	1:0.5
XG/CL/LB	XG, CL and LB	1:0.5:1
XG/CL/LBOL	XG, CL and LBOL	1:0.5:1
XG/CL/LBST	XG, CL and LBST	1:0.5:1
XGAC/CL	XGAC and CL	1:0.5
XGAC/CL/LB	XGAC, CL and LB	1:0.5:1
XGAC/CL/LBOL	XGAC, CL and LBOL	1:0.5:1
XGAC/CL/LBST	XGAC, CL and LBST	1:0.5:1

**Table 2:** Adsorption capacity of materials for degraded 5w40 motor oil.

		^a^ q_e_ ± SD, g/g	
Material		Degraded 5w40 Motor Oil	
	1 g	2 g	3 g
XG/CL	34.49 ± 1.60	35.29 ± 2.73	39.58 ± 3.57
XG/CL/LB	26.19 ± 1.94	27.03 ± 4.01	31.09 ± 6.57
XG/CL/LBOL	33.10 ± 0.34	36.35 ± 1.79	46.80 ± 3.43
XG/CL/LBST	26.82 ± 2.26	32.47 ± 2.35	36.70 ± 1.78
XGAC/CL	34.97 ± 2.08	43.53 ± 4.11	45.73 ± 3.90
XGAC/CL/LB	21.89 ± 2.32	23.99 ± 1.78	25.44 ± 3.30
XGAC/CL/LBOL	27.11 ± 0.90	27.89 ± 1.72	34.96 ± 1.37
XGAC/CL/LBST	29.99 ± 4.49	36.10 ± 3.99	39.31 ± 4.42

^a^ Data are expressed as the mean ± standard deviation (n = 3).

**Table 3:** Density, porosity, and the porous area of the studied materials.

Material	^a^ Density, g/cm^3^	^a^ Porosity, %	^b^ Pore Area, %
XG/CL	0.034 ± 0.011	90.21 ± 3.12	55.20
XG/CL/LB	0.028 ± 0.004	54.06 ± 1.23	40.94
XG/CL/LBOL	0.021 ± 0.010	98.25 ± 2.47	62.18
XG/CL/LBST	0.048 ± 0.002	84.97 ± 2.34	37.60
XGAC/CL	0.028 ± 0.0024	71.91 ± 0.93	50.55
XGAC/CL/LB	0.035 ± 0.0032	77.15 ± 1.45	49.66
XGAC/CL/LBOL	0.064 ± 0.0013	96.56 ± 3.11	43.77
XGAC/CL/LBST	0.068 ± 0.0027	72.94 ± 2.97	59.76

^a^ Data are expressed as the mean ± standard deviation (n = 3). ^b^ Pore area was determined from SEM micrographs using ImageJ 1.53 e software.

**Table 4:** DVS data of studied materials.

		BET Data	
Material	^a^ Sorption Capacity, % d.b.	^a^ Surface Area, m^2^/g	^a^ Monolayer, g/g
XG/CL	25.59 ± 2.00	169.25 ± 1.17	0.048 ± 0.020
XG/CL/LB	20.56 ± 1.69	143.62 ± 3.55	0.040 ± 0.040
XG/CL/LBOL	23.91 ± 3.77	170.44 ± 2.54	0.048 ± 0.010
XG/CL/LBST	18.35 ± 3.38	129.14 ± 5.90	0.036 ± 0.040
XGAC/CL	24.37 ± 1.45	172.80 ± 6.27	0.049 ± 0.030
XGAC/CL/LB	16.96 ± 1.98	163.55 ± 2.76	0.046 ± 0.021
XGAC/CL/LBOL	20.93 ± 2.03	154.22 ± 9.85	0.043 ± 0.024
XGAC/CL/LBST	19.63 ± 3.33	143.48 ± 0.63	0.040 ± 0.017

^a^ Data are expressed as the mean ± standard deviation (n = 3).

**Table 5:** Nominal compressive stress, strain and compressive elastic modulus values of materials.

Material	Compressive Nominal Stress, kPa	Strain, %	Compressive Elastic Modulus, kPa	R^2^
XGAC/CL	211.88 ± 10.30	88.46 ± 0.27	10.29 ± 0.47	0.999
XGAC/CL/LB	194.95 ± 9.47	64.93 ± 0.50	7.34 ± 0.39	0.992
XGAC/CL/LBOL	242.70 ± 8.52	78.43 ± 0.42	5.11 ± 0.63	0.994
XGAC/CL/LBST	173.49 ± 11.33	100.11 ± 0.09	1.24 ± 0.07	0.981

**Table 6:** Zeta potential values of the additives.

Sample	Zeta Potential, mV
CL	16.85 ± 3.01
LB	−31.06 ± 0.70
LBOL	−21.67 ± 0.90
LBST	−14.19 ± 0.06

**Table 7:** Determined kinetic parameters for adsorption of degraded 5w40 motor oil.

Material	PFO Model						PSO Model					
	1 g Oil		2 g Oil		3 g Oil		1 g Oil		2 g Oil		3 g Oil	
	k_1_, min^−1^	R^2^	k_1_, min^−1^	R^2^	k_1_, min^−1^	R^2^	k_2_ × 10^−4^, g/min × g	R^2^	k_2_ × 10^−4^, g/min × g	R^2^	k_2_ × 10^−4^, g/min × g	R^2^
XG/CL	−0.04	0.8425	−0.04	0.8491	−0.04	0.8477	1.80	0.9985	1.18	0.9977	1.89	0.9993
XG/CL/LB	−0.03	0.9231	−0.03	0.8501	−0.04	0.7097	4.26	0.9991	3.92	0.9971	3.06	0.9998
XG/CL/LBOL	−0.03	0.4641	−0.04	0.7338	−0.04	0.8787	3.09	0.9985	2.48	0.9989	0.60	0.9889
XG/CL/LBST	−0.04	0.8386	−0.03	0.9398	−0.04	0.8350	7.10	0.9999	1.66	0.9981	0.85	0.9560
XGAC/CL	−0.04	0.8496	−0.05	0.8491	−0.03	0.9511	1.26	0.9980	9.95	0.9977	0.44	0.9889
XGAC/CL/LB	−0.04	0.8396	−0.04	0.8394	−0.04	0.8441	4.84	0.9995	4.48	0.9998	2.44	0.9983
XGAC/CL/LBOL	−0.03	0.4609	−0.04	0.8356	−0.04	0.8443	6.65	0.9995	4.13	0.9996	1.27	0.9955
XGAC/CL/LBST	−0.03	0.4633	−0.04	0.8449	−0.03	0.8951	7.07	0.9997	1.76	0.9986	1.05	0.9909

**Table 8:** Adsorption parameters determined from different isotherm models for adsorption of degraded motor oil.

Materials	Henry		Langmuir				Freundlich			Temkin		
	K_H_, L/g	R^2^	q_max_, g/g	K_L_, L/g	R_L_	R^2^	n	K_F_, L/g	R^2^	A_T_, L/g	B_T_, J/mol	R^2^
XG/CL	0.083	0.8923	38.70	0.54	0.16	0.6253	13.90	28.09	0.8395	24.63	2.65	0.9337
XG/CL/LB	0.077	0.9207	30.14	0.48	0.17	0.6035	11.82	20.72	0.8411	31.99	2.41	0.5402
XG/CL/LBOL	0.232	0.9656	45.52	0.17	0.37	0.7177	5.21	19.15	0.8120	4.67	7.50	0.8164
XG/CL/LBST	0.253	0.9484	44.74	0.07	0.59	0.8988	3.30	10.42	0.9707	0.67	9.83	0.9222
XGAC/CL	0.203	0.8601	50.40	0.15	0.41	0.9088	5.37	21.12	0.9673	7.25	7.44	0.9662
XGAC/CL/LB	0.060	0.9829	26.10	0.33	0.23	0.9075	10.57	16.86	0.9949	20.43	2.23	0.9780
XGAC/CL/LBOL	0.136	0.9305	35.31	0.18	0.36	0.6610	6.23	16.83	0.9094	12.45	4.92	0.7988
XGAC/CL/LBST	0.180	0.9540	44.25	0.10	0.50	0.9335	4.56	15.34	0.9913	29.53	7.51	0.9924

## Data Availability

The data presented in this study are available in the article.

## Supplementary Materials

The following supporting information can be downloaded at: https://www.mdpi.com/article/10.3390/polym16152225/s1, Figure S1: The compression properties of materials: (A) stress–strain profiles; (B) linear dependence of stress–strain profiles used to evaluate the compression elastic moduli); (C) values of maximum sustained compression (dark cyan columns) and of compressive strength (red columns); and (D) values of elastic modulus. All values were calculated as the average of at least three individual tests ± standard deviations. Figure S2a: Experimental data fitted to the PFO kinetic model. The linear graphs were obtained from the plot of ln (q_e_ − q_t_) against time. Figure S2b: Experimental data fitted to the PSO kinetic model. Linear graphs were obtained from the plot of t/q_t_ against time. Table S1: χ^2^, Δq, RMSE values for degraded motor oil adsorption (PFO kinetic model). Table S2: χ^2^, Δq, RMSE values for degraded motor oil adsorption (PSO kinetic model). Figure S3: Linear Henry, Langmuir, Freundlich and Temkin isotherms for the adsorption of degraded 5w40 motor oil on the studied materials.
