# Supplementary material for: A Green Approach to Oil Spill Mitigation: New Hybrid Materials for Wastewater Treatment
Source: Polymers (Basel). 2024 Aug 5;16(15):2225. doi: 10.3390/polym16152225 (PMC11314923; doi:10.3390/polym16152225)
Supplement: Supplementary file 1 [file polymers-16-02225-s001.zip › polymers-3105447-supplementary.pdf]

Supplementary Materials

Degraded motor oil adsorption study

The equilibrium sorption capacity (q_e, g/g), the quantity of modified oil retained within a specific time interval (q_t, g/g), and the efficiency of used oil removal (R %) were determined using the following equations:

$q_e = \frac{W_1 - W_0}{W_0}$, g/g (S1)

$q_t = \frac{W_t - W_0}{W_0}$, g/g (S2)

$R = \frac{W_1 - W_0}{W_0} \times 100$, % (S3)

where W_0 (g) and W_1 (g) represent the initial weight of the material and its weight after the sorption process, respectively. W_t (g) represents the material weight at different time intervals.

Kinetic study

The linear equations for studied kinetic models:

PFO model: $\ln (q_e - q_t) = \ln q_e - k_1 t$ (S4)

PSO model: $q_t = \frac{q_e^2 k_2 t}{1 + k_2 q_e t}$ (S5)

where k_1 (min^{-1}) and k_2 (g/min x mg) represent the rate constants of PFO and PSO kinetic models. The linear graphs were obtained from the plot of $\ln (q_e - q_t)$ against t and t/q_t against t, respectively.

Statistical analysis

Nonlinear analysis chi-square test, $\chi^2 = \sum \frac{(qexp - qcalc)^2}{qcalc}$ (S6)

Normalized standard deviation, $\Delta q = \sqrt{\frac{\sum[\frac{qexp - qcalc}{exp}]^2}{N-1}}$ (S7)

Residual root-mean-square error, $RMSE = \sqrt{\frac{\sum_{i=1}^{n} (qexp - qcalc)^2}{N}}$ (S8)

Isotherm study

The linear equation corresponding to each studied isotherm model:

Henry isotherm model: $q_e = k_H C_e$ (S9)

Langmuir isotherm model: $\frac{1}{q_e} = \frac{1}{q_{max}} + \frac{1}{K_L q_{max} C_e}$ (S10)

$R_L = \frac{1}{1+K_L C_i}$ (S11)

Freundlich isotherm model: $\log q_e = \log K_F + \frac{1}{n} \log C_e$ (S12)

Temkin isotherm model: $q_e = B_T \ln A_T + B_T \ln C_e$ (S13)

where K_H (L/g) represents the Henry constant, and is related to the sorption capacity. K_L (L/g) represents the Langmuir constant, K_F (L/mg) and n are constants of the Freundlich adsorption isotherm, while B_T (J/mol) and A_T (L/g) represent the Temkin constants. Further analysis of the Langmuir model was performed using the equilibrium parameter, R_L, and its value indicates the favorability of the adsorption process.

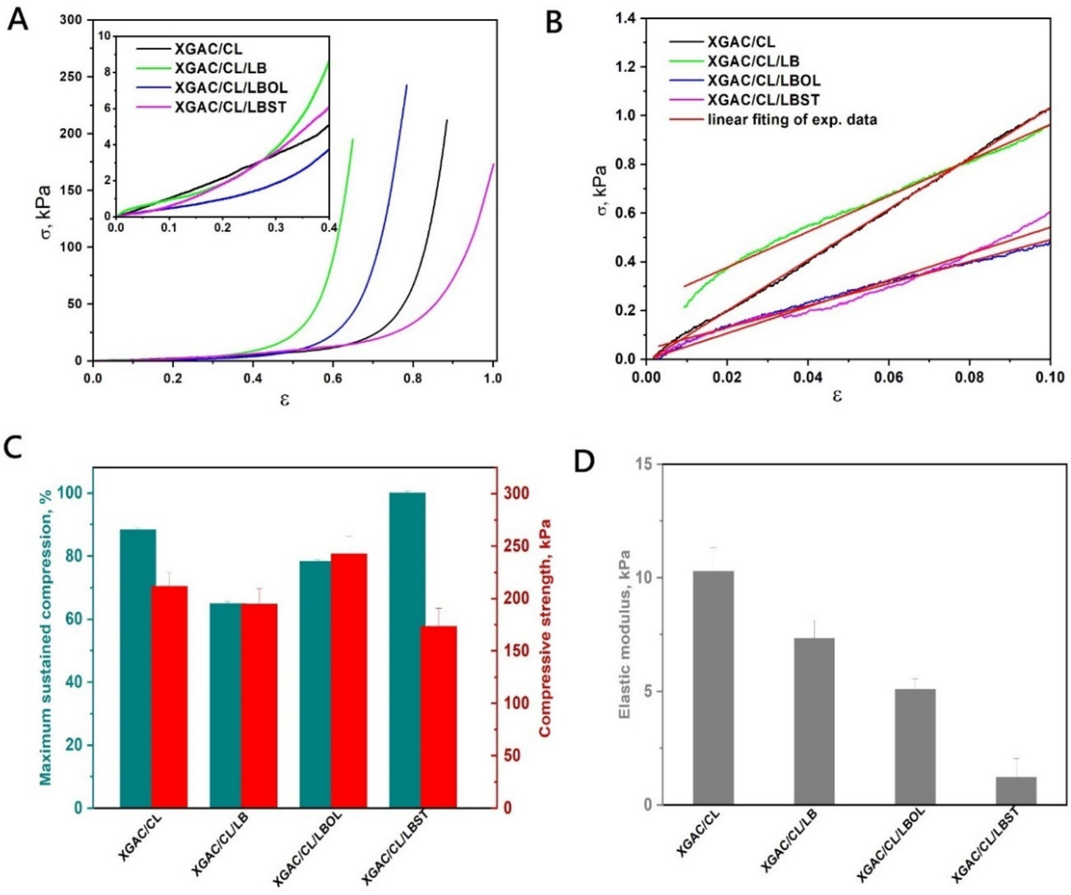

Figure S1. The compression properties of materials: (A) stress-strain profiles; (B) linear dependence of stress-strain profiles used to evaluate the compression elastic moduli); (C) values of maximum sustained compression (dark cyan columns) and of compressive strength (red columns); and (D) values of elastic modulus. All values were calculated as the average of at least three individual tests ± standard deviations.

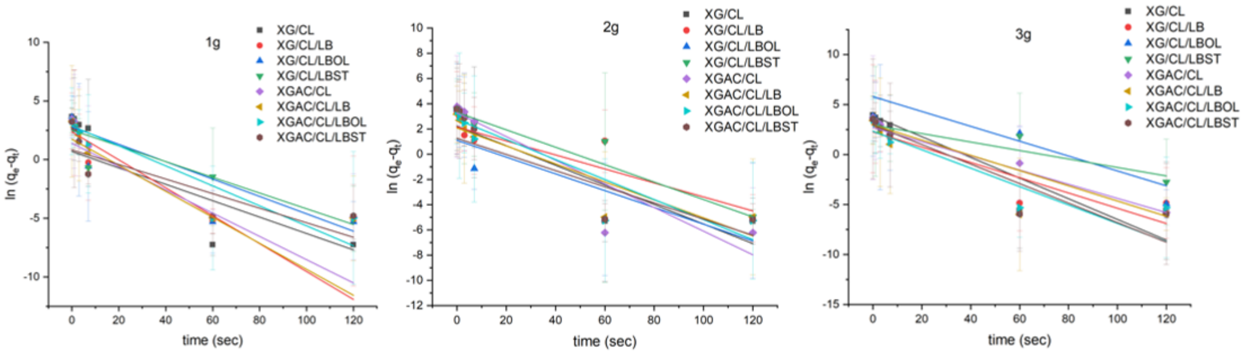

Figure S2a. Experimental data fitted to PFO kinetic model. The linear graphs were obtained from the plot of ln (qe – qt) against time.

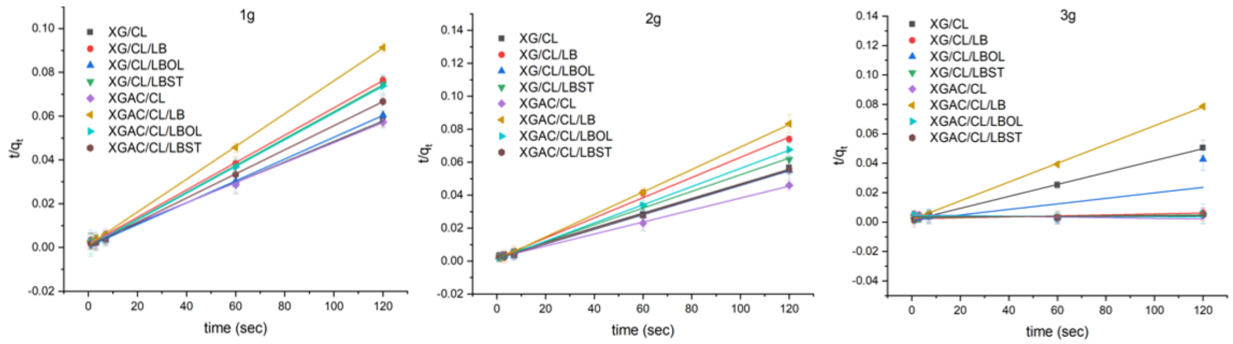

Figure S2b. Experimental data fitted to PSO kinetic model. Linear graphs were obtained from the plot of t/qt against time.

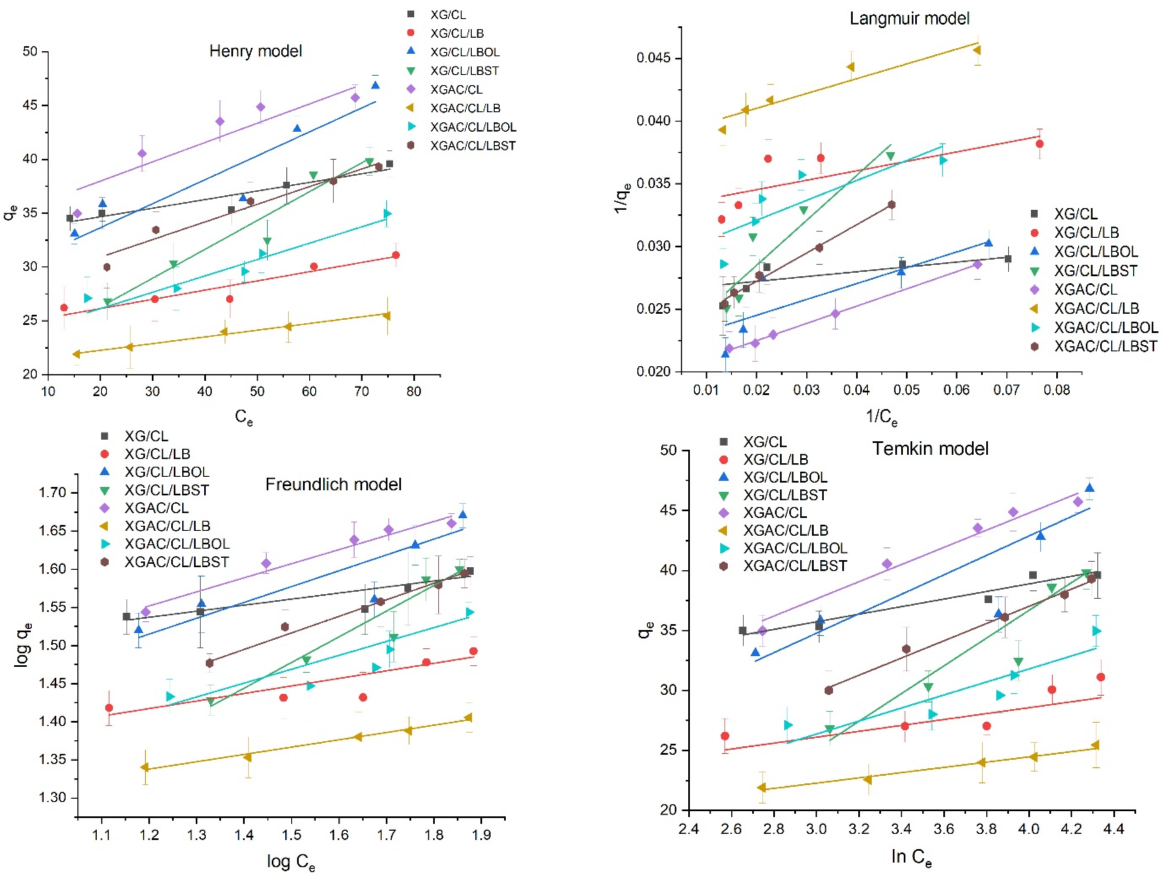

Figure S3. Linear Henry, Langmuir, Freundlich and Temkin isotherms for the adsorption of degraded 5w40 motor oil on the studied materials.

Table S1. $\chi2$, Δq, RMSE values for degraded motor oil adsorption (PFO kinetic model).

Material	Initial amount of oil, g	$\chi2$	Δq	RMSE
XG/CL	1	4.60	0.65	15.51
	2	148.00	1.18	25.70
	3	267.72	5.89	82.43
XG/CL/LB	1	325.39	1.23	20.92
	2	53.21	0.35	10.61
	3	65.05	1.39	26.02
XG/CL/LBOL	1	2.69	0.23	5.65
	2	101.64	1.15	25.74
	3	3.10	0.37	10.90
XG/CL/LBST	1	169.62	1.53	24.56
	2	128.94	1.31	31.92
	3	0.59	0.59	7.15
XGAC/CL	1	30.13	0.30	23.94
	2	126.21	0.40	31.87
	3	1.01	0.07	8.35
XGAC/CL/LB	1	71.56	0.40	14.62
	2	47.02	0.37	14.49
	3	4.43	0.17	5.98
XGAC/CL/LBOL	1	27.75	0.32	17.15
	2	7.34	0.19	10.66
	3	0.07	0.02	6.55
XGAC/CL/LBST	1	24.81	0.03	15.22
	2	28.20	0.30	18.63
	3	4.45	0.15	9.44

Table S2. $\chi 2$, Δq, RMSE values for degraded motor oil adsorption (PSO kinetic model).

Material	Initial amount of oil, g	$\chi 2$	Δq	RMSE
XG/CL	1	0.28	0.04	7.51
	2	2.19	0.11	5.66
	3	6.88	0.17	9.39
XG/CL/LB	1	3.46	0.15	5.99
	2	1.56	0.11	6.16
	3	11.46	0.23	9.59
XG/CL/LBOL	1	8.38	0.19	9.23
	2	8.64	0.19	9.59
	3	10.91	0.19	15.18
XG/CL/LBST	1	8.17	0.22	11.68
	2	12.87	0.22	20.52
	3	5.46	0.16	6.38
XGAC/CL	1	2.10	0.11	10.92
	2	7.10	0.17	16.65
	3	0.85	0.07	7.89
XGAC/CL/LB	1	2.09	0.13	3.59
	2	0.01	0.01	0.39
	3	0.04	0.02	5.32
XGAC/CL/LBOL	1	0.01	0.01	4.27
	2	0.01	0.02	2.08
	3	0.02	0.02	2.19
XGAC/CL/LBST	1	4.86	0.17	9.52
	2	6.29	0.18	12.78
	3	9.59	0.20	12.91
